# Mirror writing and cortical hypometabolism in Parkinson’s disease

**DOI:** 10.1371/journal.pone.0279007

**Published:** 2022-12-14

**Authors:** Mayumi Shinohara, Kayoko Yokoi, Kazumi Hirayama, Shigenori Kanno, Yoshiyuki Hosokai, Yoshiyuki Nishio, Toshiyuki Ishioka, Mika Otsuki, Atsushi Takeda, Toru Baba, Masashi Aoki, Takafumi Hasegawa, Akio Kikuchi, Wataru Narita, Etsuro Mori, Kyoko Suzuki

**Affiliations:** 1 Department of Behavioral Neurology and Cognitive Neuroscience, Tohoku University Graduate School of Medicine, Sendai City, Miyagi, Japan; 2 Department of Health and Welfare Science, Faculty of Sports Science, Sendai University, Shibata-machi, Shibata-gun, Miyagi, Japan; 3 Department of Occupational Therapy, Yamagata Prefectural University of Health Sciences, Yamagata City, Yamagata, Japan; 4 Department of Radiological Sciences, School of Health Sciences, International University of Health and Welfare, Otawara City, Tochigi, Japan; 5 Department of Psychiatry and Neurology, Tokyo Metropolitan Matsuzawa Hospital, Setagaya-ku, Tokyo, Japan; 6 Department of Occupational Therapy, Saitama Prefectural University, Koshigaya City, Saitama, Japan; 7 Faculty of Health Sciences, Hokkaido University, Sapporo City, Hokkaido, Japan; 8 Department of Neurology, National Hospital Organization Sendai-Nishitaga Hospital, Sendai city, Miyagi, Japan; 9 Department of Cognitive & Motor Aging, Tohoku University Graduate School of Medicine, Sendai City, Miyagi, Japan; 10 Department of Neurology, Tohoku University Graduate School of Medicine, Sendai City, Miyagi, Japan; 11 Department of Behavioral Neurology and Neuropsychiatry, Osaka University United Graduate School of Child Development, Suita City, Oosaka, Japan; Policlinico Riuniti of Foggia: S.C. Neurologia Ospedaliera, ITALY

## Abstract

Mirror writing (MW) is the production of individual letters, words, or word strings in the reverse direction. Parkinson’s disease (PD) is a progressive neurodegenerative disorder, and high MW rates have been reported in patients with PD. Thus, the present study sought to identify the factors that cause MW in patients with PD. We examined the frequency of MW in patients with PD and investigated the area of the brain where such frequency inversely correlates with reduced regional cerebral metabolic rates of glucose (rCMRglc). We also examined whether this area satisfied the motor and visual monitoring hypotheses of MW that have been presented in previous studies. Thirty-six subjects with idiopathic PD and 23 healthy controls were included in the study. We asked the participants to write down words, numerals, and sentences from left to right using their dominant and non-dominant hands. Patients with PD underwent an 18F-fluorodeoxyglucose positron emission tomography scan to measure the rCMRglc. Neither the patients with PD nor the healthy subjects exhibited MW in the use of the right hand. In the use of the left hand, MW occurred in 15 of the 36 patients with PD, but in none of the healthy controls. The right intraparietal sulcus was identified as the area where rCMRglc was inversely correlated with the number of left–right reversed characters. Previous functional imaging studies have suggested that the right superior parietal cortex and intraparietal sulcus play an important role in recognizing left–right reversed letters. Therefore, dysfunction in the intraparietal sulcus may hinder the recognition of left–right reversed characters, resulting in MW. Consequently, our findings in patients with PD are consistent with the visual-monitoring hypothesis of MW.

## Introduction

Mirror writing (MW) refers to the production of individual letters, words, or word strings in reverse direction [1]. When placed adjacent to a mirror, these letters or words can be read in the normal direction [2]. Anecdotes of deliberate MW have been reported in the biographies of famous people, including Leonardo da Vinci [2], and many children write backward spontaneously during the development of literacy [3]. MW can occur as an unintentional error in adults who write using their nondominant hand. MW is observed more frequently in people who are innately lefthanded or those who are in a right-to-left writing culture [4]. The Japanese language was traditionally written either vertically from top to down or horizontally from right to left. However, while left to right writing was strongly recommended when writing horizontally in the 1950s, right to left writing is rarely used today. In addition, writing horizontally is more common than writing vertically. Balfour et al. [1] classified MW in adults into partial MW, when individual letters alone were reversed, and complete MW, when all words or word strings were reversed. Furthermore, they reported that partial MW occurred relatively often (17.5%) in patients who had experienced a stroke; partial MW was also found to occur in healthy adults, although with a lower incidence (6.9%). In contrast, complete MWs are rare. MW has been reported in stroke patients with lesions on the left or right hemisphere, but only when writing with the nondominant hand [1]. Moreover, patients with stroke-induced MW often do not realize that they are writing the mirror images of letters, and thus do not self-correct the activity [5].

Tashiro, Matsumoto, Hamada, and Moriwaka [6] reported a high rate of MW in patients with Parkinson’s disease (PD); this included both partial and complete types of MW. Few patients self-corrected their MW, similar to patients with stroke-induced MW [6]. According to Otsuki [7], when patients with PD were asked to write using their nondominant hand, MW was found in 75.0% of the group not receiving pharmacotherapy and in 58.8% of the group receiving pharmacotherapy, which are higher than the frequencies reported by Balfour et al. [1] and also higher than the rate of stroke patients in Japan (12.9%) [8]. Therefore, MW may be more likely to occur in patients with PD than in healthy adults and stroke patients.

According to Critchley [2], there are two hypotheses for MW, namely the motor and the visual hypotheses. The motor hypothesis asserts that the most natural movement of one hand is the mirror image of the movement of the other hand: the dominant hand primarily performs the correct writing movement; thus, the natural writing movement for the nondominant hand is the mirror image of the correct writing movement. To write letters accurately with the nondominant hand, the natural mirror image movement must be suppressed, and the individual must focus on performing a different movement to write correct letters. MW is produced when there is a problem with such control. Based on this motor hypothesis, when MW arises from a neurological disorder, reduced functioning may be found in the brain area involved in controlling the action of the nondominant hand. The visual hypothesis states that it is more difficult to distinguish text flipped horizontally (left-right) than vertically (top-bottom). In other words, individuals who write mirror image letters exhibit incomplete psychological adjustment of horizontally but not vertically reversed images. Wada et al. [5] noted that patients with MW were less likely to notice mirror images of letters, and that MW appeared to involve visual and motor factors. They hypothesized that damaged visual monitoring resulted in an impaired ability to recognize that the letters being written, as well as the completed letters, were left–right reversed; therefore, self-correction of MW was rare. Based on the visual monitoring hypothesis, the lesion associated with MW is predicted to be present in the area required to monitor mirror images.

In patients with PD, the functioning of the cerebral cortex is impaired, which may cause various cognitive dysfunctions. By performing a correlation analysis between the regional cerebral metabolic rates of glucose (rCMRglcs) at rest and poor cognitive function, we can determine the cerebral region associated with such cognitive dysfunction [9, 10]. In the present study, we examined the frequency of MW in patients with PD, searched for areas of the brain for which rCMRglc inversely correlates with MW frequency, and examined whether such brain areas satisfied the above-mentioned motor and visual monitoring hypotheses of MW.

## Material and methods

### Participants

This study included 36 patients with idiopathic PD and 23 healthy control participants with corrected visual acuity better than 20/70 in both eyes. The results of PD patients were compared with those of healthy controls recruited from the community and matched for age, sex, and educational level. Table 1 shows the demographic and clinical characteristics of the two groups. All participants were innately right-handed (the Laterality Quotient of the Edinburgh Handedness Inventory was 100 in all patients, and no one had a history of switching their handedness). All were also native speakers of Japanese who wrote vertically from top to down or horizontally from left to right in their daily lives, and did not write in any other direction. Patients with PD had significantly lower scores on the Mini–Mental State Examination, significantly shorter backward digit span in the Wechsler memory Scale-Revised, and significantly shorter forward and backward tapping spans, compared with controls. It is known that upper limb dystonia disturbs handwriting [11, 12]. In this study, however, none of the patients with PD or healthy control had dystonia.

**Table 1:** Demographic and clinical characteristics of the PD patients and healthy controls.

	PD (n = 36)	Control (n = 23)	*p* value^a^
Age, mean (SD), year	67.6 (6.7)	67.4 (6.0)	0.898
Sex (female/male), n	20/16	15/8	0.461
Education, mean (SD), year	12.0 (2.3)	12.7 (2.0)	0.216
Visual acuity, median (range)	0.8 (0.4–2.0)		
MMSE, mean (SD); max. 30	27.6 (1.9)	28.5 (1.9)	0.077
Digit span, mean (SD), n			
forward	5.4 (0.9)	6.0 (0.9)	0.028
backward	3.9 (0.9)	4.5 (0.7)	0.003
Tapping span (SD), n			
forward	5.1 (0.9)	6.0 (0.9)	0.001
backward	4.6 (0.8)	5.0 (0.6)	0.01
Disease duration, mean (SD), year	8.2 (4.9)		
Onset age, mean (SD), year	59.6 (6.8)		
Daily levodopa equivalent dosage, mean (SD), mg	566.7 (288.7)		
Hoehn and Yahr, median (range)	3.0 (2.0–4.0)		
UPDRS motor part, mean (SD)	19.5 (7.7)		

PD: Parkinson’s disease, MMSE: Mini–Mental State Examination

UPDRS: Unified Parkinson’s Disease Rating Scale, SD: standard deviation.

^a^ t test was used, except for sex ratio (chi-square test) and visual acuity (Mann–Whitney U test).

The diagnosis of PD was made by board-certified neurologists in accordance with the UK PD Society Brain Bank criteria [13]. The patients’ motor symptoms were evaluated using Hoehn and Yahr staging [14] and the Unified Parkinson’s Disease Rating Scale Part III [15]. The inclusion criteria were as follows: 1) age 55–75 years at study entry, 2) age ≥40 years at disease onset, and 3) Hoehn and Yahr stage 1–3. The exclusion criteria were as follows: 1) any complications due to other neurological or psychiatric diseases and 2) any magnetic resonance imaging evidence of focal brain lesions. All patients were treated with anti-parkinsonian drugs.

All participants provided written informed consent after they and their family members were given a detailed description of the study, which was approved by the ethical committee of Tohoku University Graduate School of Medicine and conducted in accordance with the Declaration of Helsinki.

### Procedures

We asked the participants to write down from left to right using their dominant and non-dominant hands (right and left hands, respectively, in all participants). The examiner read around one word in *hiragana*, two short sentences combining *kanji* and *hiragana*, and the numbers from 1 to 10, for a total of 26 characters. Afterwards, participants were instructed to write one specific part in *kanji* and another part in *hiragana*. No feedback was provided regarding the accuracy or inaccuracy of the written characters. Reversed characters (left–right reversed) were included in the analysis. The participants who wrote one or more reversed characters conformed the MW group, and participants who did not write reversed characters constituted the group without MW. The presence or absence of differences between the groups was examined for all the demographic and clinical characteristics assessed in this study.

Each patient with PD underwent ^18^F-fluorodeoxyglucose positron emission tomography scans using a previously described protocol [9]. Positron emission tomography data were analyzed using the SPM software (version 12; Wellcome Department of Cognitive Neurology, London, UK). All images were normalized to the positron emission tomography template with nonlinear warping algorithms, reconstructed into 2-mm^3^ isotropic voxels, and smoothed with a 10-mm Gaussian kernel. Proportional scaling was used to reduce between-subject variation in the global metabolic rate.

The numbers of reversed characters were entered as covariates of interest in the correlation analyses for PD patients, with the aim of identifying regions that exhibited reduced metabolism in association with MW. Potential confounding factors associated with rCMRglc, including age, sex, levodopa equivalent daily dose, Unified Parkinson’s Disease Rating Scale Part III score, Mini–Mental State Examination score, forward and backward digit span, and forward and backward tapping span—were introduced as covariates. Furthermore, significant differences between the groups in demographic and clinical characteristics were included in our analysis, with the values of such items as confounding factors. Because the correlation analysis was confined to regions that showed hypometabolism in PD patients, relative to healthy controls, the positron emission tomography data obtained from the PD patients were contrasted with data obtained from 14 age-, sex-, and education-matched healthy elderly control participants without poor vision or cognitive impairment (7 women, 7 men; mean age, 64.0±4.2 years; mean educational attainment, 12.3±2.5 years; mean Mini–Mental State Examination score, 29.1±1.3). A resulting map with a liberal statistical threshold (*p* <0.05, uncorrected) was used as the mask image in the correlation analysis. The statistical threshold was set at an uncorrected *p* < 0.001 at the voxel level and 20 voxels at the cluster level.

## Results

**Fig 1:**
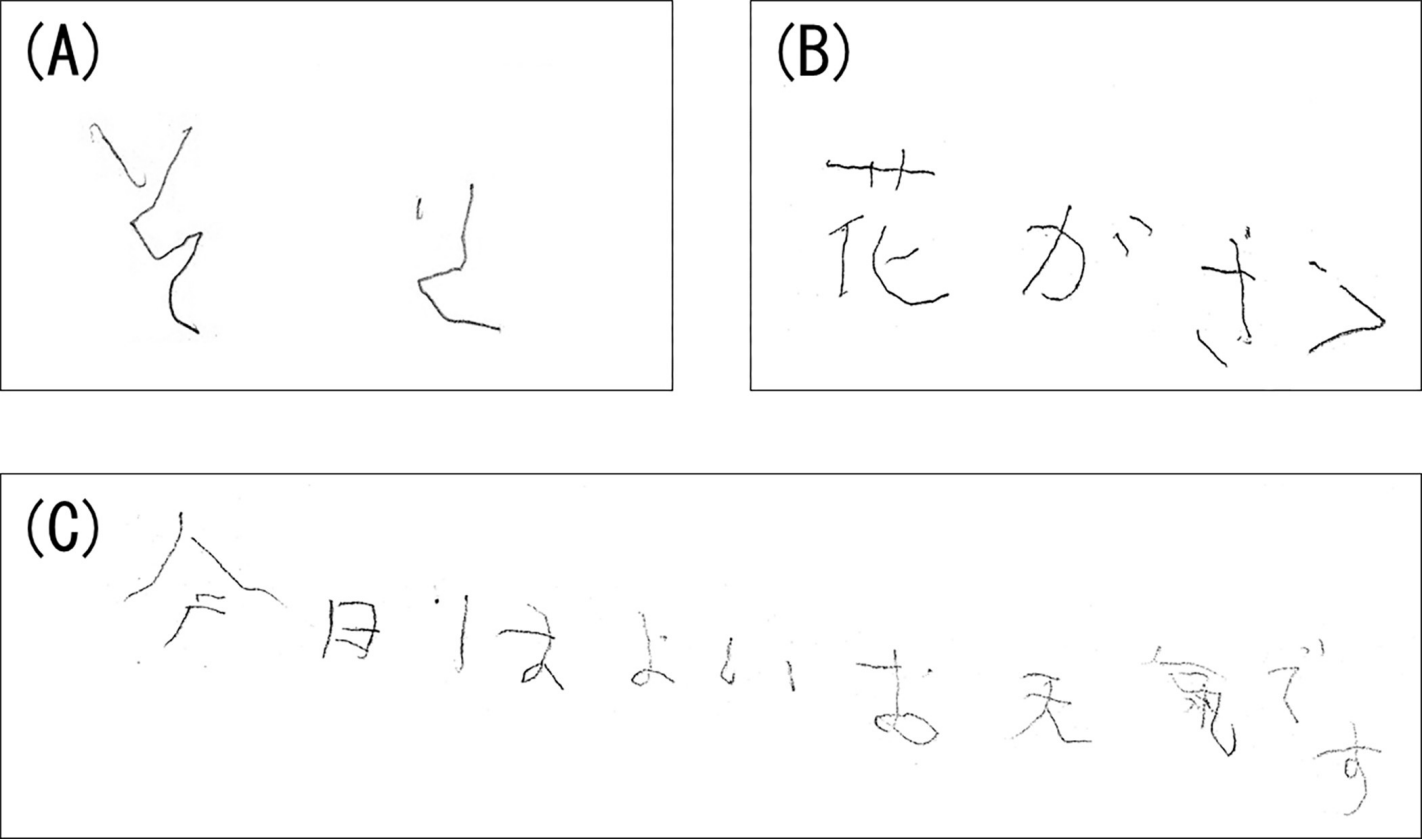
Example of mirror writing in a PD patient. (A)Task: Writing the word, “そら”. “ら” was reversed. (B) Task: Writing the sentence, “花がさく”. “く” was reversed. (C) Task: Writing the sentence, “今日はよいお天気です”. “今” was reversed.

Neither PD patients nor healthy controls exhibited MW when the right hand was used. When the left hand was used, MW occurred in 15 of the 36 patients with PD, but in none of the healthy controls. All instances of MW were partial. Fig 1 presents an example of a PD patient’s mirror writing. Only four patients self-corrected their MW. However, none of the four patients were able to correct the reversed characters immediately after they were written. All patients reacted in one of two ways: 1) they corrected MW after long contemplation after they had completed the writing task; or 2) they continued to write, then stopped and corrected the erroneous character before completing the writing task.

**Fig 2:**
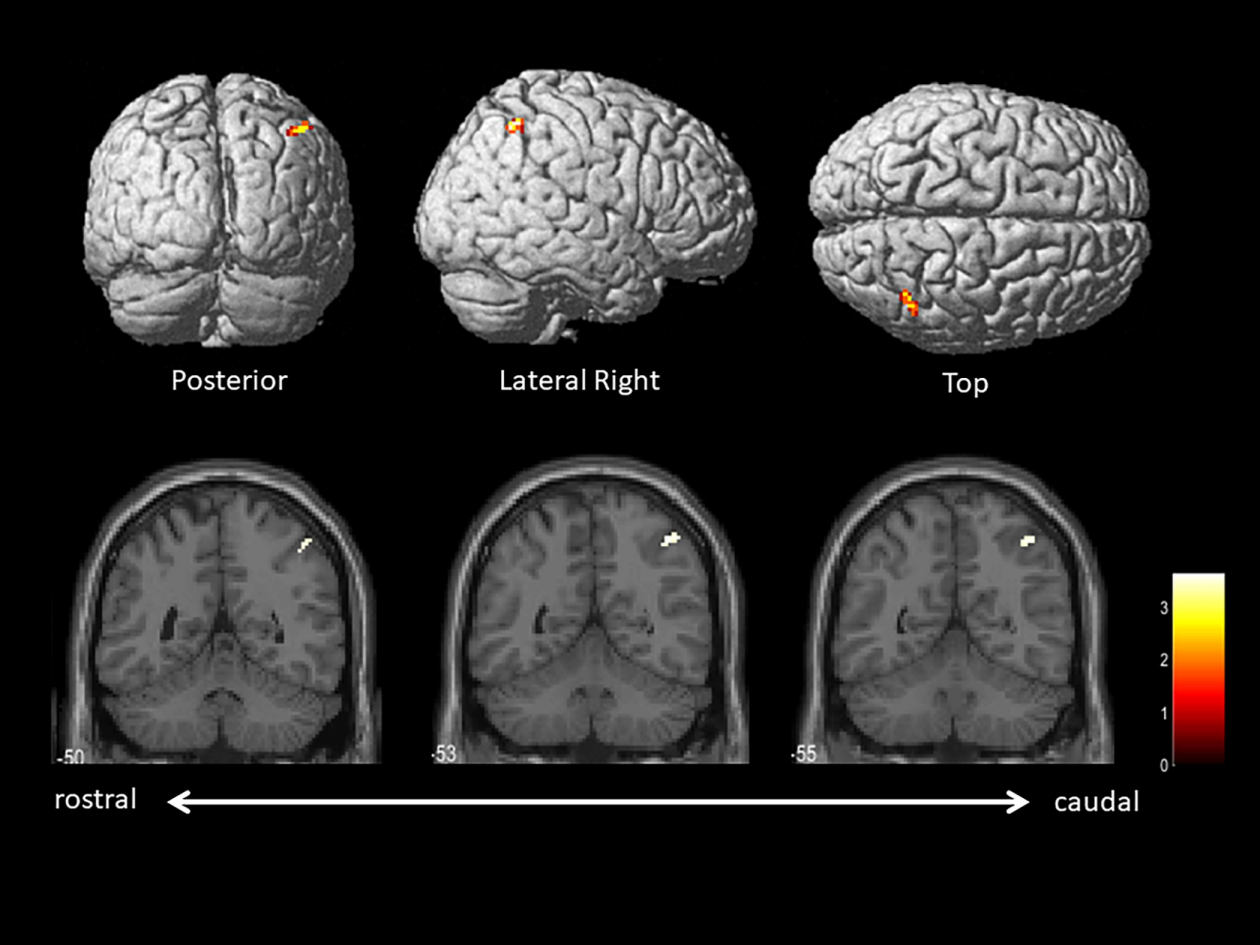
Brain regions in which the resting CMRglc was negatively correlated with the number of left–right reversed characters. The statistical threshold was set at an uncorrected *p*<0.001 at the voxel level and 20 voxels at the cluster level. Additional covariates were age, sex, levodopa equivalent daily dose, Unified Parkinson’s Disease Rating Scale Part III score, Mini–Mental State Examination score, forward and backward digit span, and forward and backward tapping spans. The results were overlaid on rendered brain (top row) or T1 single-subject template (bottom row) from SMP 12.

The median frequency of reversed characters per patient was 1 (range, 1–3). There were no significant differences in the demographic and clinical characteristics between PD patients with and without WM (Table 2). Fig 2 shows the brain regions in which the resting CMRglc was negatively correlated with the number of left-right reversed characters. The right intraparietal sulcus was identified as the area where the rCMRglc was inversely correlated with the number of left–right reversed characters (coordinates x y z: 42–54 52, z score = 3.23, and cluster size: 36). There were no other regions where resting CMRgls was significantly correlated with the numbers of left-write reversed characters.

**Table 2:** Demographic and clinical characteristics of the PD patients with mirror writing and PD patients without mirror writing.

	PD patients with MW (n = 15)	PD patients without MW (n = 21)	*p* value^a^
Age, mean (SD), year	68.9 (5.9)	66.7 (7.2)	0.347
Education, mean (SD), year	11.3 (1.7)	12.4 (2.5)	0.152
Sex (female/male), n	10/5	10/11	0.320
Visual acuity, median (range)	0.8 (0.8–1.0)	0.8 (0.4–2.0)	0.072
MMSE, mean (SD); max. 30	27.2 (2.2)	27.9 (1.6)	0.275
Digit span, mean (SD), n			
forward	5.5 (0.5)	5.4 (1.1)	0.776
backward	3.9 (0.6)	3.9 (1.0)	0.975
Tapping span (SD), n			
forward	4.9 (0.8)	5.2 (0.9)	0.298
backward	4.5 (1.0)	4.6 (0.7)	0.600
Disease duration, mean (SD), year	6.9 (3.4)	9.2 (5.6)	0.162
Onset age, mean (SD), year	61.9 (6.0)	57.9 (6.9)	0.074
Daily levodopa equivalent dosage, mean (SD), mg	550.5 (289.6)	578.3 (307.7)	0.780
Hoehn and Yahr, median (range)	3.0 (2.5–4.0)	2.5 (2.0–4.0)	0.263
UPDRS motor part, mean (SD)	19.7 (8.4)	19.4 (7.4)	0.895
Laterality of motor symptoms (Right/Left), n	8/7	14/7	0.499

PD, Parkinson’s disease; MW, mirror writing; MMSE, Mini-Mental State Examination; UPDRS, Unified Parkinson’s Disease Rating Scale; SD, standard deviation.

^a^t test was used, except for sex ratio and laterality of the motor symptoms (Fisher’s Exact test) and visual acuity (Mann–Whitney U test).

## Discussion

The present study was performed to examine the frequency of MW in patients with PD and to identify the areas of the brain in which rCMRglc inversely correlated with MW frequency. Subsequently, examinations were performed to determine whether the identified brain region was consistent with one or both of the following hypotheses: 1) the motor hypothesis, according to which the control mechanism to suppress MW is damaged, and 2) the visual monitoring hypothesis, which asserts that it is difficult for patients to recognize that they write mirror image letters because of damage to the mechanism required for visual monitoring.

In the present study, MW was observed in 41.7% of patients with PD. It has been noted that the underlying mechanisms differ between complete and partial types of MW [1]. However, in the present study, all instances of MW were partial, as only individual characters were reversed; therefore, the frequency may constitute an indicator of a consistent level of impairment.

There were no significant differences between PD patients with MW and those without MW with respect to age, education, sex, visual acuity, disease duration, onset age, daily levodopa equivalent dosage, PD motor severity, laterality of motor symptoms, general cognition, general attention, and executive function. MW might occur independently of those demographic and clinical characteristics, perceptions and cognitive function factors, although care must be taken when interpreting those results since the analysis was performed on a small number of patients and only used for between-group comparisons.

The right intraparietal sulcus area was identified as the region in which rCMRglc was inversely correlated with the number of left–right reversed characters. Alivisatos and Petrides [16] showed in a positron emission tomography-based study that the right posterosuperior parietal cortex was active during the task of discriminating normal letters from those flipped horizontally (left–right reversed). In a study using functional magnetic resonance imaging, Poldrack, Desmond, Glover, and Gabrieli [17] showed that the bilateral superior parietal cortex was active when reading reversed strings of letters; however, only the activity of the right superior parietal cortex was reduced in association with proficiency. In another functional magnetic resonance imaging study, Goebel, Linden, Lanfermann, Zanella, and Singer [18] found an association between reading left–right reversed strings of letters and the intraparietal sulcus. In addition, Dong, Fukuyama, Honda, Okada, Hanakawa et al. [19] conducted a functional magnetic resonance imaging study of Japanese individuals who read left–right reversed strings of kana characters. This study showed that the activity of the area close to the medial wall of the intraparietal sulcus of the right superior parietal lobule was significantly correlated with behavioral performance. The findings of these functional imaging studies suggest that the right superior parietal cortex and intraparietal sulcus play important roles in the recognition of left-right reversed letters.

Therefore, the correlation between the incidence of MW and reduction of rCMRglc in the right intraparietal sulcus in patients with PD may partly explain the mechanism by which this decline in brain function causes difficulties with mirror image perception. Specifically, this decline causes difficulty in recognizing that characters are left-right reversed, both while writing them and after completion of writing, resulting in MW. Consequently, the mechanism of MW in patients with PD appears to be consistent with the visual monitoring hypothesis.

This study had some limitations. Participants in the present study exhibited only partial MW; therefore, the area of the brain associated with complete MW remains unclear. Future studies should include patients with PD who exhibit complete MW to clarify this point. Furthermore, in the present study, we did not ask participants to perform mirror-image discrimination; therefore, we could not fully confirm whether the patients exhibited an impaired ability to discriminate mirror images. However, Natsopoulos, Bostantzopoulou, Katsarou, Grouios, and Mentenopoulos [20] previously described impaired immediate memory of mirror image patterns in patients with PD. Future research should be conducted to reveal the mechanisms of mirror image discrimination ability in patients with PD.

As noted in the introduction, the frequency of MW is influenced by differences in handedness and writing direction [4]. However, in this study, we were only able to examine native Japanese speakers who were innately right-handed and write from left to right in their daily life. To the best of our knowledge, previous MW studies on patients with PD are limited to Japanese [6, 7] and Chinese [21] populations. In the future, if the presence or absence of MW in non-dominant hand will be investigated during clinical examination and MW in left-handed patients or patients with different writing direction styles will be examined in combination with imaging study like in this study, it will help understand the influence of these factors on MW and the differences in the underlying brain functional regions.
